# Supplementary material for: In vivo High-Content Screening in Zebrafish for Developmental Nephrotoxicity of Approved Drugs
Source: Front Cell Dev Biol. 2020 Jul 10;8:583. doi: 10.3389/fcell.2020.00583 (PMC7366291; doi:10.3389/fcell.2020.00583)
Supplement: TABLE S3.PDF — Supplementary Table 3.pdf. [file Table_3.PDF]

Table S3. Main ATC D-level groups provoking renal developmental toxicity.

ATC D-level description	ATC code D-level	Chemical name
**Dihydropyridine derivatives**	C08CA	Amlodipine
		**Felodipine**
		**Isradipine**
		Nicardipine hydrochloride
		**Nifedipine**
		Nimodipine
		**Nitrendipine**
		**Lacidipine**
		**Nilvadipine**
		**Cilnidipine**
		Benidipine hydrochloride
**HMG CoA reductase inhibitors**	C10AA	**Simvastatin**
		**Lovastatin**
		Pravastatin
		**Fluvastatin sodium salt**
		**Atorvastatin**
**Fibrates**	C10AB	**Clofibrate**
		Bezafibrate
		**Gemfibrozil**
		**Fenofibrate**
		Ciprofibrate
**Imidazole and triazole derivatives**	D01AC	**Sulconazole nitrate**
		Bifonazole
		**Oxiconazole nitrate**
		**Sertaconazole nitrate**
**Corticosteroids, moderately potent (group II)**	D07AB	**Flumethasone**
		Flumethasone pivalate
		**Desonide**
		**Alclometasone dipropionate**
		**Clocortolone pivalate**
**Corticosteroids, potent (group III)**	D07AC	**Fluocinonide**
		**Fluocinolone acetonide**
		Flurandrenolide
		**Diflorasone diaceate**
		Amcinonide
		Prednicarbate
**Imidazole derivatives**	G01AF	Clotrimazole
		Econazole nitrate
		**Isoconazole**
		**Tioconazole**
		**Butoconazole nitrate**

**Glucocorticoids**	H02AB	**Betamethasone**<br>**Dexamethasone acetate**<br>**Methylprednisolone, 6-alpha**<br>**Prednisolone**<br>Prednisone<br>**Triamcinolone**<br>**Hydrocortisone base**<br>**Cortisol acetate**<br>Cortisone<br>Rimexolone<br>Deflazacort
**Acetic acid derivatives and related substances**	M01AB	**Indometacin**<br>Sulindac<br>Tolmetin sodium salt dihydrate<br>**Zomepirac sodium salt**<br>**Diclofenac sodium**<br>Etodolac<br>**Fentiazac**<br>Acemetacin<br>**Ketorolac tromethamine**<br>**Aceclofenac**<br>Bufexamac
**Propionic acid derivatives**	M01AE	**S-(+)-ibuprofen**<br>**Naproxen**<br>Ketoprofen<br>**Fenoprofen calcium salt dihydrate**<br>**Fenbufen**<br>Suprofen<br>**Flurbiprofen**<br>**Indoprofen**<br>Tiaprofenic acid<br>**Oxaprozin**
**Benzimidazole derivatives**	P02CA	**Mebendazole**<br>Tiabendazole<br>**Albendazole**<br>Flubendazole<br>**Fenbendazole**

Drugs affecting renal development as evaluated by quantitative measurements are highlighted in bold. Abbreviation: ATC, Anatomical Therapeutic Chemical Classification system.
