# Supplementary material for: In vivo High-Content Screening in Zebrafish for Developmental Nephrotoxicity of Approved Drugs
Source: Front Cell Dev Biol. 2020 Jul 10;8:583. doi: 10.3389/fcell.2020.00583 (PMC7366291; doi:10.3389/fcell.2020.00583)
Supplement: TABLE S4.PDF — Supplementary Table 4.pdf. [file Table_4.PDF]

Table S4. Additional compounds with adverse effects on pronephros development.

E-level description	ATC D-level description	ATC code D-level
Omeprazole	Proton pump inhibitors	A02BC
Dicyclomine hydrochloride	Synthetic anticholinergics, quaternary ammonium compounds	A03AA
Warfarin	Vitamin K antagonists	B01AA
**Proscillaridin A**	**Scilla glycosides**	**C01AB**
**Amiodarone hydrochloride**	**Antiarrhythmics, class III**	**C01BD**
Metaraminol bitartrate	Adrenergic and dopaminergic agents	C01CA
Midodrine hydrochloride	Adrenergic and dopaminergic agents	C01CA
Canrenone	Aldosterone antagonists	C03DA
Pentoxifylline	Purine derivatives	C04AD
**Suloctidil**	**Other peripheral vasodilators**	**C04AX**
**Diltiazem hydrochloride**	**Phenylalkylamine derivates**	**C08DB**
**Lidoflazine**	**Other non-selective calcium channel blockers**	**C08EX**
Valsartan	Angiotensin II antagonists, plain	C09CA
**Irbesartan**	**Angiotensin II antagonists, plain**	**C09CA**
**Ciclopirox ethanolamine**	**Other antifungals for topical use**	**D01AE**
**Isotretinoin**	**Retinoids for treatment of acne**	**D10BA**
**Norgestimate**	**Progestogens and estrogens, fixed combinations**	**G03AA**
**Progesterone**	**Pregnen (4) derivatives**	**G03DA**
**Danazol**	**Antigonadotropins and similar agents**	**G03XA**
Gestrinone	Antigonadotropins and similar agents	G03XA
**Fludrocortisone acetate**	**Mineralocorticoids**	**H02AA**
Methicillin sodium	Beta-lactamase resistant penicillins	J01CF
Sulbactam	Beta-lactamase inhibitors	J01CG
Tazobactam	Beta-lactamase inhibitors	J01CG
Cefotaxime sodium salt	Third-generation cephalosporins	J01DD
Troleandomycin	Macrolides	J01FA
Streptomycin sulfate	Streptomycins	J01GA
**Nalidixic acid sodium salt**	**Other quinolones**	**J01MB**
Oxolinic acid	Other quinolones	J01MB
**Amphotericin B**	**Antibiotics**	**J02AA**
**Miconazole**	**Imidazole derivates**	**J02AB**
Dapsone	Drugs for treatment of lepra	J04BA
Delavirdine	Non-nucleoside reverse transcriptase inhibitors	J05AG
Raltitrexed	Folic acid analogues	L01BA
**Carmofur**	**Pyrimidine analogues**	**L01BC**
Epirubicin hydrochloride	Anthracyclines and related substances	L01DB
Topotecan	Other antineoplastic agents	L01XX
**Flutamide**	**Anti-androgens**	**L02BB**
**Nilutamide**	**Anti-androgens**	**L02BB**

Exemestane	Aromatase inhibitors	L02BG
**Leflunomide**	**Selective immunosuppressants**	**L04AA**
Azathioprine	Other immunosuppressants	L04AX
Thalidomide	Other immunosuppressants	L04AX
**Phenylbutazone**	**Butylpyrazolidines**	**M01AA**
**Piroxicam**	**Oxicams**	**M01AC**
**Meloxicam**	**Oxicams**	**M01AC**
**Mefenamic acid**	**Fenamates**	**M01AG**
Nimesulide	Other anti-inflammatory and antirheumatic agents, non-steroids	M01AX
Auranofin	Gold preparations	M01CB
**Etofenamate**	**Antiinflammatory preparations, non-steroids for topical use**	**M02AA**
**Felbinac**	**Antiinflammatory preparations, non-steroids for topical use**	**M02AA**
Cisatracurium besylate	Other quaternary ammonium compounds	M03AC
**Diflunisal**	**Salicylic acid and derivatives**	**N02BA**
Valproic acid	Fatty acid derivatives	N03AG
Entacapone	Other dopaminergic agents	N04BX
**Fluspirilen**	**Diphenylbutylpiperidine derivatives**	**N05AG**
**Pimozide**	**Diphenylbutylpiperidine derivatives**	**N05AG**
Asenapine maleate	Diazepines, oxazepines and thiazepines	N05AH
**Isocarboxazid**	**Monoamine oxidase inhibitors, non-selective**	**N06AF**
**Disulfiram**	**Drugs used in alcohol dependence**	**N07BB**
Isoflupredone acetate	n/a	n/a
**Nocodazole**	**n/a**	**n/a**
**Retinoic acid**	**n/a**	**n/a**
**GBR 12909 dihydrochloride**	**n/a**	**n/a**
Pregnenolone	n/a	n/a
Parthenolide	n/a	n/a
**Mevastatin**	**n/a**	**n/a**
Tranilast	n/a	n/a
Hexestrol	n/a	n/a
**Cycloheximide**	**n/a**	**n/a**
Butylparaben	n/a	n/a
**Methiazole**	**n/a**	**n/a**
Homosalate	n/a	n/a
**Clonixin Lysinate**	**n/a**	**n/a**
**Ethoxzolamide**	**n/a**	**n/a**
**Benzoxiquine**	**n/a**	**n/a**
**Clioquinol**	**Hydroxyquinoline derivatives**	**P01AA**
Diloxanide furoate	Dichloracetamide derivatives	P01AC
**Halofantrine hydrochloride**	**Other antimalarials**	**P01BX**
Ivermectin	Avermectines	P02CF
Altrenogest	n/a	n/a

**Flunixin meglumine**	**n/a**	**n/a**
**Oxibendazole**	**n/a**	**n/a**
**Parbendazole**	**n/a**	**n/a**
Flunisolide	Corticosteroids	R01AD
Mometasone furoate	Corticosteroids	R01AD
**Salmeterol**	**Selective beta-2-adrenoreceptor agonists**	**R03AC**
Beclomethasone dipropionate	Glucocorticoids	R03BA
Budesonide	Glucocorticoids	R03BA
Fluticasone propionate	Glucocorticoids	R03BA
Meclozine dihydrochloride	Piperazine derivatives	R06AE
Astemizole	Other anthistamines for systemic use	R06AX

Drugs with a strong effect on pronephros development as judged by quantitative analysis are highlighted in bold. Abbreviation: ATC, Anatomical Therapeutic Chemical Classification system.
